# Gene-Directed Enzyme/Prodrug Therapy of Rat Brain Tumor Mediated by Human Mesenchymal Stem Cell Suicide Gene Extracellular Vesicles In Vitro and In Vivo

**DOI:** 10.3390/cancers14030735

**Published:** 2022-01-31

**Authors:** Miroslav Tibensky, Jana Jakubechova, Ursula Altanerova, Andrea Pastorakova, Boris Rychly, Ladislav Baciak, Boris Mravec, Cestmir Altaner

**Affiliations:** 1 Institute of Physiology, Faculty of Medicine, Comenius University, 81372 Bratislava, Slovakia; miroslav.tibensky@fmed.uniba.sk (M.T.); boris.mravec@fmed.uniba.sk (B.M.); 2 Institute of Experimental Endocrinology, Biomedical Research Center, Slovak Academy of Sciences, 84505 Bratislava, Slovakia; 3 Cancer Research Institute, Biomedical Research Center, Slovak Academy of Sciences, 84505 Bratislava, Slovakia; jana.jakubechova@ousa.sk; 4 Department of Stem Cell Preparation, St. Elisabeth Cancer Institute, 84505 Bratislava, Slovakia; ursula.altanerova@ousa.sk; 5 Institute of Medical Biology, Genetics and Clinical Genetics, Faculty of Medicine, Comenius University, 81108 Bratislava, Slovakia; andrea.pastorakova@fmed.uniba.sk; 6 Alpha Medical, Ltd., 82606 Bratislava, Slovakia; boris.rychly@gmail.com; 7 Central Laboratories, Slovak University of Technology, 81237 Bratislava, Slovakia; 6baciak@gmail.com

**Keywords:** rat glioblastoma, gene-directed enzyme prodrug therapy, mesenchymal stem cells, suicide gene extracellular vesicles, yCD::UPRT-MSC-EVs, intranasal application, curative effect

## Abstract

**Simple Summary:**

Extracellular vesicles— exosomes—secreted by human mesenchymal stem/stromal cells are able to cross the blood–brain barrier and internalize glioblastoma cells. We prepared exosomes possessing a gene message, the product of which is able to convert nontoxic 5-fluorocytosine to cytotoxic drug 5-fluorouracil. Such therapeutic exosomes administered intranasally, intraperitoneally, or subcutaneously to rats bearing intracerebral glioblastoma cells inhibited their growth. The treatment cured a significant number of animals.

**Abstract:**

MSC-driven, gene-directed enzyme prodrug therapy (GDEPT) mediated by extracellular vesicles (EV) represents a new paradigm—cell-free GDEPT tumor therapy. In this study, we tested the efficacy of yeast cytosine deaminase::uracilphosphoribosyl transferase (yCD::UPRT-MSC)-exosomes, in the form of conditioned medium (CM) to inhibit the growth of C6 glioblastoma cells both in vitro and in vivo. MSCs isolated from human adipose tissue, umbilical cord, or dental pulp engineered to express the yCD::UPRT gene secreted yCD::UPRT-MSC-exosomes that in the presence of the prodrug 5-fluorocytosine (5-FC), inhibited the growth of rat C6 glioblastoma cells and human primary glioblastoma cells in vitro in a dose-dependent manner. CM from these cells injected repeatedly either intraperitoneally (i.p.) or subcutaneously (s.c.), applied intranasally (i.n.), or infused continuously by an ALZET osmotic pump, inhibited the growth of cerebral C6 glioblastomas in rats. A significant number of rats were cured when CM containing yCD::UPRT-MSC-exosomes conjugated with 5-FC was repeatedly injected i.p. or applied i.n. Cured rats were subsequently resistant to challenges with higher doses of C6 cells. Our data have shown that cell-free GDEPT tumor therapy mediated by the yCD::UPRT-MSC suicide gene EVs for high-grade glioblastomas represents a safer and more practical approach that is worthy of further investigation.

## 1. Introduction

Glioblastoma multiform (GBM) grade IV is one of the most malignant types of brain tumors. Current treatment options for patients with GBM include surgical resection, chemotherapy, and radiotherapy, but despite therapeutic advances, the prognosis for most patients is poor, with a median survival time of only 14.6 months. Complications such as the blood–brain barrier (BBB), which forms a physiological barrier for brain drug delivery [1], but also cancer stem cells (CSCs) [2] that are capable of infiltrating the brain parenchyma, are one of the possible reasons for the failure of current GBM therapies. Chemotherapeutic agents are capable of killing most cells in a tumor, except for CSCs that survive due to their relatively high resistance to drugs and a low rate of proliferation. Although CSCs make up only a small fraction of cells in the tumor microenvironment, their property of being immortal is sufficient to allow tumor recurrence. Furthermore, tumor cells under chemotherapy or radiotherapy release extracellular vesicles (EVs) that are responsible for the establishment of drug-resistant cell subpopulations [3]. Additional major disadvantages of standard systemic therapies are that these drugs are not specifically targeted for tumors. Therefore, novel therapeutic modalities are required that can attack CSCs and can specifically target tumors without developing tumor resistance.

Mesenchymal stromal/stem cells (MSCs) are multipotent stem cells with tumor homing capacity, thereby becoming a part of the tumor microenvironment. This specific ability of MSCs inspired us to develop a treatment based on gene-directed enzyme/prodrug therapy mediated by MSCs engineered to express suicide genes [4]. Specifically, these engineered MSCs have integrated transcriptional active yeast cytosine deaminase (CD)::uracil phosphoribosyl transferase fusion gene (yCD::UPRT) from a provirus. The enzyme catalyzes the conversion of noncytotoxic 5-fluorocytosine (5-FC) into cytotoxic 5-fluorouracil (5-FU). We found that genetically modified MSCs maintain the ability to migrate to tumors and inhibit tumor growth in the presence of the prodrug 5-FC. The mechanism of MSC homing to a tumor is currently not fully understood. Several studies have shown that the chemoattraction of MSCs into a tumor is dependent upon the cytokine chemokine receptor interaction between SDF-1/CXCR4, SCF-c-Kit, HGF/c-Met, VEGF/VEGFR, PDGF/PDGFr, and MCP-1/CCR2 [5,6]. In addition, growth factors (IGF-1E), angiogenic factors (βFGF, HIF1α), chemokines (CCL5, CCL2, CXCL12, and CCL22), and cytokines (TNFα, TGFβ IL-1β, and IL-8) can also be included. Several scientific teams, including us, have shown that following systemic administration of MSCs leads to their migration into the tumor microenvironment. Recently, therapeutic roles of mesenchymal stem cell-derived extracellular vesicles in cancer were reviewed [7].

The prodrug gene therapy mediated by MSCs has effectively inhibited the growth of human colon carcinoma [4], melanoma [8], and prostate carcinoma [9] in nude mice. Furthermore, a positive therapeutic effect of the autologous and human yCD::UPRT-AT-MSCs cells was proven in autochthonous prostate adenocarcinoma in TRAMP mice, which spontaneously develop an aggressive prostate cancer [10]. Adipose tissue-derived MSCs (AT-MSCs) engineered to express the yCD::UPRT gene were shown to induce a curative therapeutic effect in a substantial number of rats with intracranial C6 glioblastoma in a preclinical model [11,12]. In all these preclinical animal models, we frequently noticed an antitumor effect, despite being unable to detect therapeutic cells in the tumor site. An explanation came when it was found that these cells release EVs, which are able to transfer mRNAs and miRNAs to another cell that can be expressed and translated in recipient cells [13,14]. MSC-derived EVs exert their therapeutic effects through a paracrine/endocrine manner in regenerative medicine [15]. Importantly, the tumor tropism of MSCs remains in secreted EVs enabling them to be used in targeted cancer gene therapy [16,17].

A new, improved treatment modality appeared with our finding that yCD::UPRT gene-transduced MSCs release EVs (yCD::UPRT-MSC-exos) carrying the mRNA of the suicide gene in their cargo. These EVs migrate to the tumor in a similar manner as MSCs, where they are internalized by the tumor cells [18,19]. Direct visual evidence for the internalization of MSC exosomes in glioma cells was shown by confocal microscopy [20]. EVs have been observed to cross an intact BBB [21] and, therefore, can be used for the treatment of brain disorders, including tumors.

In this study, we report the inhibition of growth of intracranial rat glioblastoma C6 by yCD::UPRT-MSC-EVs–EVs that were administered intraperitoneally, intranasally, and subcutaneously, together with prodrug 5-FC. Furthermore, repeated administration of conditioned medium (CM) from yCD::UPRT-MSCs has led to strong inhibition of tumor growth and a significant number of animals appearing to be cured.

## 2. Materials and Methods

### 2.1. Cell Cultures

The isolation of human MSCs from adipose tissue (AT-MSCs) was done as previously described [4]. Human MSCs, derived from dental pulp stem cells (DP-MSCs) or umbilical cord (UC-MSCs), were isolated from obtained tissue fragments adhered to plastic tissue culture dishes [22]. All MSCs were cultivated in a complete culture medium DMEM low glucose (1 g/L) supplemented with 5% human platelet extract (PE) at 37 °C in a humidified atmosphere with 5% CO_2_.

Transduction of MSCs with the yCD::UPRT gene was performed as previously described [23]. Briefly, MSCs were infected with the retrovirus prepared on helper cells, and transduced cells were selected for resistance to G418 (0.6 mg/mL). A pure population of transduced cells was then expanded in low glucose (1 g/L) DMEM supplemented with 5% PE. Rat glioblastoma C6 cells were cultured in DMEM supplemented with 5% FCS and an antibiotic–antimycotic mix (Life Technologies, Gaithersburg, MD, USA) in a humidified atmosphere and 5% CO_2_ at 37 °C.

### 2.2. Preparation of yCD::UPRT-MSC-CM-exos/5-FC Conjugate

To prepare a conditional medium of the yCD::UPRT gene transduced MSCs, the semiconfluent cell cultures were washed with phosphate-buffered saline and cultured over 24 h in a medium without any growth supplements (2 mL/10^6^ cells). The conditional medium was centrifuged at 500 g for 5 min to remove any debris, filtered through a 0.2 μm syringe filter, and stored at −80 °C. The concentration of EVs in the range of 40–120 nM was 10^8–10^/mL set by NanoSight. The addition of 5-FC in a concentration of 100 μg/mL made a mixture that is the basis for the formation of the conjugate. When this mixture was left for 2 h at ambient temperature or was incubated at 37 °C for 10 min, the conjugates of 5-FC and mRNA encoding yCD::UPRT fused suicide gene in extracellular vesicles were formed. The conjugate is stable in biological activity at ambient temperature and is enriched with extracellular vesicles in a range below 30 nM.

### 2.3. Cell Growth Assessment Using the IncuCyte Live Cell Monitoring System

The cell growth was monitored by “real time in vitro micro-imaging” using the IncuCyte system (Essen Instruments, Ann Arbor, MI, USA). Most cell viability data obtained from the IncuCyte system were in good accordance with data obtained by subjecting the plates to the CellTiter 96 Aqueous One Solution Cell Proliferation Assay (Promega, Madison, WI, USA).

### 2.4. Animal Experiments

This study was performed on seven weeks old adult male CDVR IGS rats purchased from Charles River (Charles River, Köln, Germany). Rats were housed four per cage and maintained under standard laboratory conditions (12 h light–dark cycle, lights on at 7:00 a.m., ambient temperature 22 ± 2 °C and 55 ± 10% humidity) and were fed by standard pelleted rat chow and tap water ad libitum. This experiment was approved by the State Veterinary and Food Administration of the Slovak Republic (Ro/2216/19/221) within the animal facility SK UCH 01017 and approved by the Ethics Committee of Biomedical Center of the Slovak Academy of Sciences in conformity with the DIRECTIVE 2010/63/EU on the protection of animals used for scientific purposes. Before all surgical procedures, anesthesia was induced in all animals with an intramuscular injection of a mixture containing ketamine (Narkamon 5%, 1.2 mL/kg b.w.) and xylazine (Rometar 2%, 0.4 mL/kg b.w.). Injection of glioblastoma C6 cells in the amount of 5 × 10^5^ or 1 × 10^6^ for corresponding experiments was performed by stereotaxic technique. This number of cells guarantees the quick onset of tumor growth. Inspection of animal behavior and body weight during the experiment was performed every day. Animals that reached excessive weight loss or uncoordinated behavior were euthanized by decapitation, and the brain was taken on histopathological analyses.

### 2.5. Stereotaxic Implantation of Cells

The intracerebral implantation of glioblastoma cells was performed using digital stereotaxic apparatus (David-Kopf Instruments, Los Angeles, CA, USA). For the intracerebral implantation of C6 cells, selected stereotaxic coordinates were chosen according to Paxinos and Watson [24] and were as follows: anteroposterior: 0.0 mm; mediolateral: 3.0 mm; vertical: 6.0 mm. Resuspended tumor cells were slowly injected in 5 µL of PBS over a 5 min period using a Hamilton syringe. The needle was left in place for 2 min and then slowly elevated from the brain over 3 min.

### 2.6. Implantation of Osmotic Pumps for Continuous Delivery of the Prodrug 5-FC or yCD::UPRT-MSC-CM

Continuous delivery of 5-FC and yCD::UPRT/MSC-CM was achieved by surgical implantation of two cannulas at the sides of the cranial bone. The cannula from the site of implantation was then connected by a tube to an osmotic pump Model 2ML2 (ALZET, Durect Corporation, Cupertino, CA, USA) filled with 2 mL of either 5-FC or yCD::UPRT/MSC-CM. The tube led from the brain into the location of the osmotic pump that was implanted subcutaneously in the back area. The rats continuously received a therapeutic or 5-FC from the osmotic pump at 5 µL/h. Pumps were left in place for two weeks and then replaced by new ones. Subsequently, after the next two weeks, the pumps were removed. The osmotic pumps were replaced and removed under anesthesia.

### 2.7. Magnetic Resonance Imaging

MRI experiments were performed on a 4.7 T horizontal scanner (Agilent, Yarnton, UK) equipped with a 400 mT/m gradient insert and DDR console. A quadrature volume coil transmitter with i.d. of 72 mm and a dual-channel, anatomically shaped surface coil receiver (both from Rapid Biomed, Rimpar, Germany) were used for multislice T2 weighted experiments (FSEMS TR/TE/NEX: 3600/40/7). Thirty 1 mm thick slices of the brain without gaps were selected to cover the whole brain lesion. The overall scan time was 13 min, and the pixel resolution was 0.16 × 0.16 mm^2^. During the experiment, animals were anesthetized with 2% isoflurane mixed with air. The respiration was monitored, and an ambient temperature was maintained at 37 °C by warm air (SA instruments, Rimpar, Germany).

### 2.8. Statistical Analysis

The Kaplan–Meier survival curves and survival times of groups of rats were compared using a log-rank test (GraphPad Prism Version 6.0 software, La Jolla, CA, USA). Differences with *p* < 0.05 were considered statistically significant.

## 3. Results

### 3.1. A Schematic Overview 

**Figure 1:**
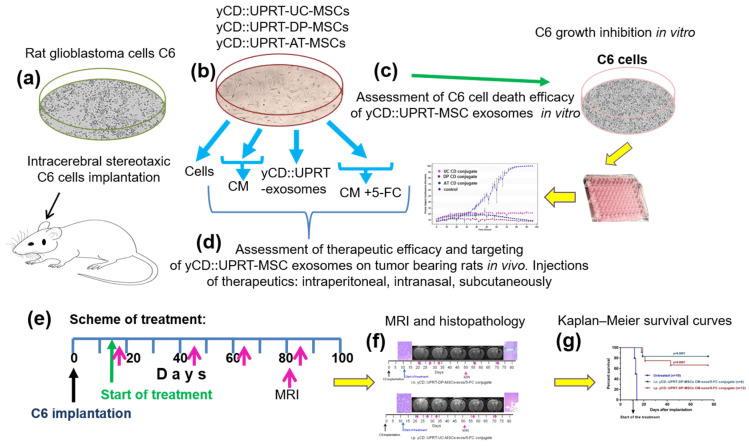
Schematic overview of steps performed in the study. (**a**) Rats were injected with a dose of 5 × 10^5^ or 1 × 10^6^ C6 cells intracerebrally by a stereotaxic technique. (**b**) Sources of therapeutic materials tested were: Cells, CM with yCD::UPRT-MSC-exosomes, and yCD::UPRT-UC-MSC-exos/5-FC conjugate from the umbilical cord, dental pulp, and adipose tissue MSCs, stably transduced with yCD::UPRT gene. (**c**) The course of growth/inhibition was monitored and evaluated by the IncuCyte system. (**d**) Therapeutic efficacy and targeting assessment of different forms of therapeutic materials applied i.n., i.p., or s.c. in rats intracerebrally bearing C6 glioblastoma (**e**) Scheme of treatment. (**f**) Evaluation of therapeutic outcomes by MRI and final histopathology (**g**) Survival of treated animals evaluated in Kaplan–Meir survival curves.

All Steps Performed in This Study Is Presented in Figure 1.

### 3.2. Sensitivity of Rat Glioblastoma C6 and Primary Human Glioblastoma Cells to the Cytotoxic Effect of yCD::UPRT-MSC-exos In Vitro

**Figure 2:**
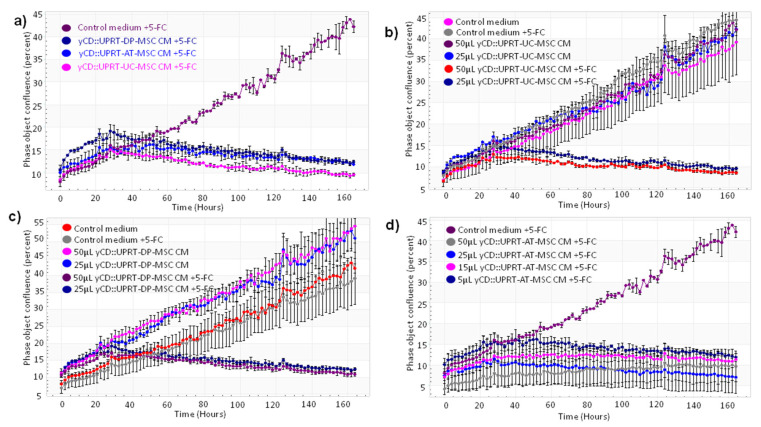
Growth curves of C6 glioblastoma cells treated with CM from various yCD::UPRT-UC-MSCs. The course of growth/inhibition was monitored by the IncuCyte system. The cell density was followed by image scanning of the culture every 2 h over 6 d. The standard deviation was calculated for each scan. (**a**) Growth inhibition of C6 cells with CM from yCD::UPRT-AT-MSCs, from yCD::UPRT-UC-MSCs, and from yCD::UPRT-DP-MSCs in the presence or absence of 5-FC. (**b**) Growth curves of HPGC cultivated in vitro and treated with CM from yCD::UPRT-UC-MSCs in the absence or presence of 5-FC. (**c**) Growth inhibition of HPGC treated with CM from yCD::UPRT-DP-MSCs in the presence or absence of 5-FC. (**d**) Growth inhibition of HPGC treated with CM from yCD::UPRT-AT-MSCs in the presence or absence of 5-FC. 5-FC, 5-fluorocytosine; CM—conditioned medium; HPGC—human primary glioblastoma cells.

Our objective was to verify the feasibility and efficacy of CM containing yCD::UPRT-exos harvested from yCD::UPRT-gene transduced human MSCs of different tissue origin to inhibit the growth of rat C6 glioblastoma cells both in vitro and in vivo. The human umbilical cord, dental pulp, and adipose tissue-derived MSCs transduced with yCD::UPRT gene integrated into cell DNA and selected for a pure population of yCD::UPRT-transduced cells were used in this study. The cells were designated yCD::UPRT-UC-MSCs, yCD::UPRT-DP-MSCs, and yCD::UPRT-AT-MSCs. All release EVs into growth medium that possess mRNA of the yCD::UPRT gene in their cargo [17]. We will refer to them here as yCD::UPRT-MSC EVs (yCD::UPRT-MSC-exos). The effect of CM from three yCD::UPRT-MSCs in the presence or absence of 5-FC was monitored by the IncuCyte system. In agreement with our previous work with other tumor cell lines, CM from all tested cells inhibited the growth of C6 cells (Figure 2a).

In order to test the susceptibility of yCD::UPRT-MSC-exos to internalize human primary glioblastoma cells (HPGC) cultivated in vitro, we generated growth curves in the absence or presence of the prodrug. CM from yCD::UPRT-DP-MSCs without 5-FC slightly supported the growth but killed all cells in the presence of 5-FC (Figure 2b). CM from yCD::UPRT-UC-MSCs without 5-FC did not influence the growth of HPGCs, while in the presence of 5-FC, a strong inhibition was observed (Figure 2c). CM with yCD::UPRT-AT-MSC-exosomes in the presence of 5-FC inhibited the growth of HPGCs efficiently (Figure 2d). The growth of treated HPGCs resulted in either stimulation or inhibition, which was expressed in a dose-dependent manner.

### 3.3. Cytotoxic Effect of yCD::UPRT-MSCs and Their EVs in Rats Bearing Glioblastoma C6 In Vivo

**Figure 3:**
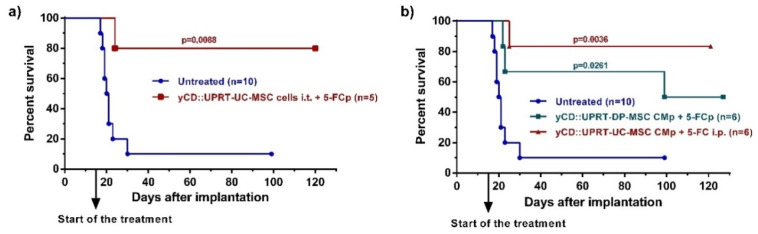
Rat glioblastoma C6 growth inhibition mediated by human yCD-UC-MSC-exosomes in vivo. Tumors were induced by 5 × 10^5^ C6 cells inoculated intracerebrally. Animals began treatment with CM from yCD-DP-MSCs and yCD-UC-MSCs applied continuously by an ALZET osmotic pump Model 2ML2 intratumorally with one exchange for a fresh pump. The prodrug (5-FC; 500 mg/kg of body weight) was delivered i.p. three times per week or continuously by an ALZET osmotic pump. Animals were killed on day 100 or 120, and their brains were inspected by histopathology. (**a**) Survival of rats bearing intracerebral C6 glioblastoma cells treated with 10^5^ yCD::UPRT-UC-MSCs implanted intracerebrally. The prodrug was delivered by an ALZET osmotic pump (**b**) Survival of rats bearing intracerebral C6 glioblastoma cells treated with yCD::UPRT-DP-MSC-CM and yCD::UPRT-UC-CM applied by an ALZET osmotic pump. Statistical significance was determined by comparison to the untreated animal group. 5-FCp, 5-fluorocytosine delivered by an osmotic pump; CMp— conditioned medium delivered by an osmotic pump.

In our previous work, we reported high levels of therapeutic effects with human adipose tissue-derived yCD::UPRT-gene transduced MSCs implanted in a growing, intracerebral C6 tumor [11]. In the same way, we administrated yCD::UPRT gene transduced human umbilical cord-derived cells yCD::UPRT-UC-MSCs, followed by 5-FC delivery by ALZET osmotic pumps. The application of intratumor applied cells led to a cure of most brain glioblastoma-bearing rats (Figure 3a). In order to determine whether the yCD::UPRT-exos present in CM are able to cure an intracerebrally located C6 tumor, we tested their therapeutic efficacy in the presence of the prodrug 5-FC in three independent experiments on C6 cell bearing rats. A dose of 5 × 10^5^ rat glioblastoma cells was chosen for the experiments and was implanted intracerebrally by stereotaxic surgery. Intracerebral injection of C6 cells resulted in the death of all rats except one after 30 days in two independent experiments. Treatment began 15 days after tumor cell implantation and started with the implantation of ALZET osmotic pumps filled with CM from yCD::UPRT-DP-MSCs and yCD::UPRT-UC-MSCs. Pumps were exchanged for fresh ones once after 12 days. The prodrug 5-FC was delivered at a dose of 500 mg/kg b.w. by repeated i.p. injections (three times per week) or continuously by ALZET osmotic pumps. The continuous injections of the prodrug 5-FC, either i.p. or by ALZET osmotic pump, was equally effective. The experiments were ended on day 100 or 120, and animals were inspected for tumors by biopsy and histopathology. We tested the therapeutic effectivity of CM from yCD::UPRT-UC-MSCs or yCD::UPRT-DP-MSCs, in two independent experiments. The data obtained from both experiments were pooled and presented as Kaplan–Meier survival graphs (Figure 3b). The percentage of surviving rats treated with CM containing yCD::UPRT-MSC-exos was in the range of 50 to 85%, regardless of the cellular origin of MSCs (UC, DP).

### 3.4. Tumoricidal Behavior of CD::UPRT-MSC-CM-exos/5-FC Conjugates

To determine whether it would be possible to use a mixture of CM with 5-FC for in vivo applications, we tested the mixture for its biological activity after it was left for several days at room temperature. We found that the mixture inhibited the growth of C6 cells in a dose-dependent manner. We named MSCs isolated from umbilical cord yCD::UPRT-UC-MSC-CM-exos/5-FC conjugate, MSCs isolated from dental pulp yCD::UPRT-DP-MSC-CM-exos/5-FC conjugate, and MSCs isolated from adipose tissue yCD::UPRT-AT-MSC-CM-exos/5-FC conjugate.

**Figure 4:**
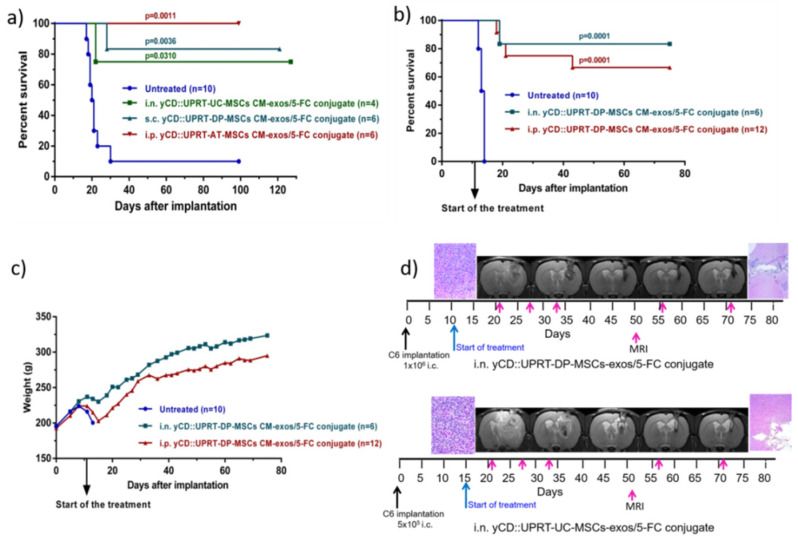
Rat glioblastoma C6 growth inhibition mediated by human yCD::UPRT-MSC-exos/5-FC conjugates in vivo. Tumors were induced by 5 × 10^5^ or 1 × 10^6^ C6 cells inoculated intracerebrally. (**a**) Survival of rats bearing 5 × 10^5^ intracerebral C6 glioblastoma cells treated with yCD::UPRT-DP-MSC-exos/5-FC conjugate, yCD::UPRT-UC-MSC-exos/5-FC conjugate, and yCD::UPRT-AT- MSC-exos/5-FC conjugate applied s.c., i.n., and i.p. (**b**) Survival of rats bearing 1 × 10^6^ intracerebral C6 glioblastoma cells treated with yCD::UPRT-DP-MSC-exos/5-FC conjugate by i.n. application (every day) and yCD::UPRT-UC-MSC-exos/5-FC conjugate applied i.p. three times per week (**c**) Course of body weights of treated and untreated animals during the experiment. (**d**) MRI examinations of treated animals during the course of treatment and final histopathological examinations. 5-FC, 5-fluorocytosine; MRI—magnetic resonance imaging.

The tumoricidal behavior of yCD::UPRT-UC-MSCs CM-exos/5-FC conjugate and yCD::UPRT-DP-MSCs CM-exos/5-FC conjugate was determined after being applied intranasally, subcutaneously, or intraperitoneally. Intraperitoneal administration of yCD::UPRT-AT-MSCs CM-exos/5-FC conjugate was tested as well. Animals were treated with conjugates applied i.p. and s.c. at a dose of 1 mL three times a week for seven weeks. The intranasal application was performed every day for seven weeks. The treatment was started 15 days after C6 cell implantation. As shown in Figure 4a, 75–100% of rats treated with conjugates from various cellular origins of MSCs (UC, DP, and AT) survived. All modalities of conjugate administration were effective as well. Conjugates also had a rapid onset of action, as shown in Figure 4a, because treatment started only two days before the first rat in the control group died. Based on these results, we chose a higher amount of intracerebral implanted glioblastoma C6 cells in the next experiment. In this experiment, the treatment started 11 days after C6 cell implantation. Rats treated intraperitoneally with yCD::UPRT-UC-MSC-exos/5-FC conjugate had a survival rate of 67%, unlike the intranasally treated group, which had a survival rate of 83% (Figure 4b). Both conjugates did not influence body weight following repeated administration (Figure 4c). The course of treatment was followed by MRI scanning and histopathological examination (Figure 4d).

### 3.5. Resistance of Rats Cured with yCD::UPRT-MSC-exos/5-FC to Reimplantation of C6 Cells

Almost all animals that survived after being treated with CM from yCD::UPRT-MSCs or yCD::UPRT-MSC-exos/5-FC conjugate regardless of the source of MSCs tissue origin and modality of treatment were reimplanted, approximately after 3–4 months, intracerebrally into an opposite hemisphere with 1 × 10^6^ C6 cells, a double dose of cells in comparison with the dose of 5 × 10^5^ that caused death in almost all animals within 30 days. The body weights of these animals were measured for 32 days after C6 cell implantation. All 17 animals survived the challenge, their body weight rose during the experiment, and histopathological examination revealed no glioblastoma cells in the brain.

## 4. Discussion

Previous preclinical studies in rats bearing intracerebral C6 glioblastoma cells have reported curative outcomes in significant numbers of animals treated with human yCD::UPRT-AT-MSCs applied intratumorally [11,12]. Release of extracellular vesicles from yCD::UPRT-AT-MSCs possessing the mRNA of a suicide gene in the cargo of EVs shifted the MSC-driven gene-directed enzyme prodrug therapy (GDEPT) to a new paradigm—cell-free tumor therapy [17,25]. In this study, we tested the feasibility and efficacy of yCD::UPRT-DP-MSC-exosomes in the form of CM to inhibit the growth of C6 glioblastoma cells using both in vitro and in vivo experiments. Three MSCs of different tissue origin isolated from human adipose tissue, umbilical cord, and dental pulp were transduced with yCD::UPRT suicide gene by retrovirus infection, which led to the integration of the yCD::UPRT gene into cell DNA and its subsequent expression. A pure population of yCD::UPRT-gene transduced MSCs were the source of EVs–yCD::UPRT-MSC-exosomes that we tested for growth inhibition of C6 glioblastoma in rats. The results achieved are in good agreement with previously published positive outcomes, when only cells were used for treatment [11,12].

EVs secreted from the suicide gene transduced MSCs reflect naïve MSCs’ characteristics. The route of administration of therapeutics to diseases localized to the brain is an important factor influencing the efficacy of therapy. For immunogenic oncolytic adenovirus encapsulated in extracellular vesicles, the intraventricular route was found to be much better than intravenous or intraperitoneal ones [26]. We also found that the therapeutic potential of yCD::UPRT-MSC-EVs was not strictly dependent on the route of administration. Their good therapeutic efficacy can be attributed to several factors, including the tumor-tropic property and the ability to bypass the BBB [21]. The potential to be injected intraperitoneally, subcutaneously, or even administered intranasally is a great advantage and evidence for their tumor tropism. The ability to invade cells and, most importantly, the production of the cytotoxic drug inside the tumor cell is a reason for their high tumor cell killing efficiency. Consequently, no side effects connected with 5-FC/5-FU therapy were observed in the treated animals. Furthermore, yCD::UPRT-MSC-EVs do not have cell therapy-associated problems, demonstrating that they are safer because they are cell-free.

Intranasal delivery provides an extraordinary approach for the treatment of intracerebral gliomas [27]. A noninvasive intranasal application route for suicide gene EVs was found to be quite effective in confirming our previous findings showing migration of dental pulp-derived MSCs labeled with iron sucrose to intracerebral glioblastoma in rats [28]. Intranasal delivery of MSC-derived extracellular vesicles seems to be a very promising way of administrating therapeutics in regenerative medicine, which has been recently comprehensively reviewed [29].

The Secretome of yCD::UPRT-MSCs represented by CM is composed of yCD::UPRT exosomes and a mixture of factors resembling the exosome’s cargo. The prodrug 5-FC is required to achieve a therapeutic effect, as we found that the addition of 5-FC to CM at ambient temperature converts the mixture to a stable conjugate named yCD::UPRT-MSC-CM-exos/5-FC. Gene-directed enzyme prodrug therapy (GDEPT) is a two-step process [30]. In the first step, the gene for yCD::UPRT is delivered to the tumor and expressed. When prodrug 5-FC is administred, it is converted by the enzyme to cytotoxic 5-FU, and by the catalytic action of UPRT is progressed to 5-FU metabolites. In the case of the exosome-based conjugate, the whole process is done in one step. Messenger RNA of yCD::UPRT gene in EVs together with prodrug is delivered to the tumor cell. In addition, because of MSC-derived exosomes, it is tumor targeted, and the process is intracellular. Glioblastoma therapy provided by yCD::UPRT-UC-MSC-CM-exos/5-FC conjugate administered i.p. and with yCD::UPRT-DP-MSC-CM-exos/5-FC administered i.n. appeared to be very efficient (Figure 4). Based on our preliminary data, the nature of yCD::UPRT-MSC-CM-exos/5-FC conjugates resemble exosomes in their activity [31]. These potent therapeutic nanoparticles were characterized by the unusual temperature stability of yCD::UPRT-MSC-CM/5-FC conjugates, sustained tumor cell death activity, tumor cell targeting, ability to cross BBB, and most importantly, the creation of the cytotoxic drug inside the tumor cell. In the past, significant in vitro and in vivo studies on the inhibition of tumor growth by protein 5-FC conjugates leading to intracellular conversion to 5-FU have been reported [32,33]. Protein conjugates of CD with 5-FC have showed effective, targeted antitumor potency while maintaining high CD enzyme activity to convert 5-FC to 5-FU [34,35].

In an agreement with previously reported induction of protective immunity by tumors expressing the CD suicide gene eliminated with 5-FC [36], we observed the same effect in C6-bearing rats treated with yCD::UPRT-MSC-EVs.

GDEPT mediated by EVs with a suicide gene represents a promising therapy for brain tumors. Furthermore, preclinical studies are needed to see whether this therapeutic approach will succeed in clinical trials. MSC-driven GDEPT clinical trials have presented promising results that warrant further investigation [37].

## 5. Conclusions

Extracellular vesicles—exosomes—secreted from yCD::UPRT-MSCs were found to be targeted to glioblastomas in rat brain whether they were applied through the noninvasive route of intranasal application or injected intraperitoneally or subcutaneously. We also found that CM supplemented with prodrug 5-FC repeatedly applied to rats bearing C6 glioblastomas led to curative outcomes in a significant number of animals. Intranasal delivery of yCD::UPRT-MSC-EVs/5-FC conjugate may represent an important noncellular therapeutic approach for the treatment of brain tumors, providing that it will be tested in the clinical trial.

## Data Availability

The data that support the findings of this study are available from the corresponding author upon reasonable request.
